# Case Report: Culture-negative Prosthetic Valve Endocarditis

**DOI:** 10.2174/1573403X19666230411151214

**Published:** 2023-10-02

**Authors:** Ignacio D. Velazquez, Kenneth K. Woo, Mohammed Siddiqui, Sion K. Roy

**Affiliations:** 1 Division of Cardiology, Harbor-UCLA Medical Center, Torrance, CA, USA

**Keywords:** Fungal endocarditis, aspergillus endocarditis, prosthetic valve endocarditis, culture-negative infective endocarditis, transesophageal echocardiograms, treatment

## Abstract

**Background:**

Prosthetic valve endocarditis can be difficult to diagnose and cause significant morbidity and mortality, especially when no culture data are available to guide therapy. Transthoracic and transesophageal echocardiograms, the studies of choice for initial endocarditis evaluation, can be less reliable due to artifact and post-surgical changes. Some less common forms of endocarditis may be difficult to culture and, due to their fastidious nature, may delay the identification of causative organisms. Given the lack of directed antimicrobial treatment, culture-negative prosthetic valve endocarditis is specifically difficult. A wide differential diagnosis is critical to make a timely diagnosis and initiate treatment.

**Case Presentation:**

We present a case of a patient presenting with dyspnea which was found to have culture-negative endocarditis requiring mitral and aortic valve replacement that ultimately was complicated with culture-negative prosthetic valve endocarditis. Identifying a culprit organism made appropriate and timely antimicrobial treatment difficult, ultimately resulting in the patient dying from endocarditis complications.

**Conclusion:**

A high index of suspicion is needed when managing infective endocarditis, especially when prosthetic valves are involved. Diagnostic accuracy of cultures and echocardiography may be reduced when dealing with prosthetic valve endocarditis; thus, alternative methods of diagnosis may be required to make a timely diagnosis of causative organisms.

## 1 INTRODUCTION

The incidence of infective endocarditis is estimated to be up to 10 per 100,000 cases per year and rising [1]. One-year mortality for infective endocarditis is estimated to be 20% in community-acquired infections but up to 73% in those with prosthetic valves [1, 2]. Unfortunately, a causative organism cannot be identified in about 10% of prosthetic valve endocarditis cases [3]. These cases are difficult to manage due to multiple challenges. First, the organisms are fastidious and lead to delays in diagnosis and treatment. Second, the diagnosis of infectious endocarditis *via* transthoracic echocardiography has diminished sensitivity (23%) and specificity (36-69%) compared to native valve endocarditis [3]. Finally, while continuing to be the gold standard diagnostic decision tool, the modified Duke criteria have a decreased diagnostic accuracy in the setting of suspected prosthetic valve infections [4]. We present a rare case of culture-negative prosthetic valve endocarditis where a diagnosis was delayed, and the patient ultimately expired.

## 2 CASE SUMMARY

**Fig. (1):**
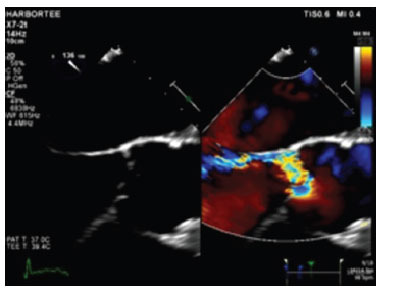
Transesophageal echocardiogram showing severe eccentric aortic regurgitation.

Our patient is a 58-year-old male with no known past medical history. He presented to the emergency room with new onset dyspnea on exertion, orthopnea, paroxysmal nocturnal dyspnea, and abdominal/lower extremity swelling following an upper respiratory tract infection. Symptoms were considered consistent with acute decompensated heart failure, and a transthoracic echocardiogram was ordered to evaluate. A transthoracic echocardiogram showed severe aortic and mitral regurgitation, a left ventricular ejection fraction of 30%, and no evidence of vegetation. There was no mention of Osler’s nodes, Janeway lesions, rash, joint pain, or splinter hemorrhage; thus, the etiology of the clinical presentation was thought to be due to decompensated chronic valvular disease. He was discharged after medical optimization but failed plans of outpatient evaluation for valve replacement, given recurrent admission for decompensated heart failure due to severe aortic and mitral regurgitation. During this admission, a transesophageal echocardiogram was performed for surgical planning, which showed two 4mm-6mm lesions on the A2 and A3 scallops of the mitral valve concerning vegetations as well as severe eccentric regurgitation of the aortic valve with prolapse of the right coronary cusp (Fig. **1**). He was placed on empiric ceftriaxone and doxycycline while being worked up for culture-negative endocarditis (repeat blood cultures and Coxiella serology) which ultimately returned negative. At this time, the patient did not meet definitive Duke criteria for infective endocarditis, and after a multidisciplinary meeting, his presentation was not thought to be consistent with infective endocarditis. The decision was made to proceed with mitral and aortic valve replacements with bio-prosthetic valves. During surgery, the valves were found to be largely normal appearing, other than a possible healed vegetation on the mitral valve. Blood cultures and intraoperative valve cultures remained negative, and the patient was discharged home with a six-week course of Ceftriaxone and Doxycycline for presumed culture-negative endocarditis.

One month post-discharge, he was seen in the cardiology clinic, where he complained of fevers/chills and was found to be hypotensive and tachycardic. He was admitted, and a subsequent transesophageal echocardiogram identified a small echo density on the prosthetic mitral valve. However, no organism was identified despite multiple blood cultures. After a multidisciplinary meeting, the patient was treated empirically with antibiotics for prosthetic valve endocarditis targeting *Staphylococcus* and *Streptococcus*, two of the most common pathogens. At this time, the patient’s clinical course was further complicated by the development of a complete heart block requiring the placement of a biventricular pacemaker/implantable cardioverter defibrillator, after which the patient was discharged home.

**Fig. (2):**
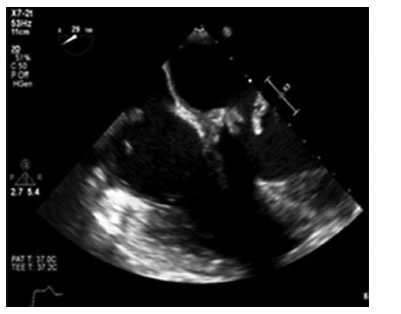
Transesophageal echocardiogram with vegetation on and dehiscence of the bioprosthetic mitral valve.

**Fig. (3):**
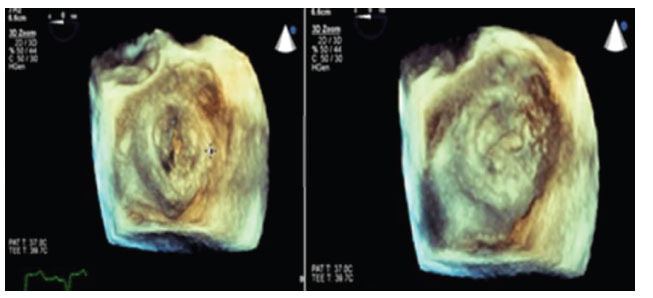
3D Transesophageal echocardiogram showing bioprosthetic mitral valve with multiple large bulky vegetations and dehiscence.

Two months later, the patient was admitted for the final time with fevers and chest pain symptoms. A physical exam was revealing for conjunctival and splinter hemorrhages, and computed tomography of his chest revealed wedge-shaped infarcts of the spleen and kidneys. Repeat transesophageal echocardiogram revealed multiple, large bulky vegetations encasing the mitral valve leaflets with extension to the aorto-mitral curtain and aortic root and valve dehiscence (Figs. **2** and **3**). Multiple blood cultures were finalized as negative; however, serum Aspergillus enzyme immunoassay and Beta-D glucan tests returned positive at 2.24 and >500, respectively and were highly concerning for disseminated Aspergillus infection. The patient was started on amphotericin B, voriconazole. Six days after being drawn, and after the final read, reported no growth, the blood cultures were reported to be growing mold. The patient was scheduled for re-do aortic and mitral valve replacement but developed focal intracerebral hemorrhage, which precluded valve surgery. His clinical condition deteriorated, and the patient expired. The family declined an autopsy.

## 3 DISCUSSION

In this case, the patient presented with culture-negative prosthetic valve endocarditis after valve replacement. It is suspected that the original valvular lesions were from a healed episode of endocarditis. The lack of organisms isolated on blood cultures may have been attributed to the empiric antibiotics he had been prescribed after his valve replacements since approximately 50% of culture-negative infective endocarditis cases have been associated with prior antibiotic therapy [5]. Nevertheless, these same studies show that fastidious organisms may account for up to 20% of cases [5]. In this case, the diagnosis may have been delayed, given he did not have a history of an immunocompromising state that would have predisposed him to fungal infection and did not meet definite Duke criteria for infective endocarditis. However, this may not have been completely surprising given that the Duke criteria are less accurate in prosthetic valve endocarditis, given its reduced sensitivity compared to native valve endocarditis [4].

As evidenced in this case, it is important to allow a broad differential in the setting of culture-negative prosthetic valve endocarditis. Although the classic teaching of evaluating for bacterial causes should not be ignored, fungal infections like Aspergillus should be considered particularly in post-surgical patients, patients with recent antibiotic exposure, or those who do not respond to initial therapy targeted at more common bacterial pathogens. Illustrating this point further, a retrospective study took 31 patients with aspergillus endocarditis compared with 331 patients with non-fungal endocarditis and found that hospital-acquired endocarditis, prosthetic valve endocarditis, presence of pseudoaneurysm or abscess, and absence of fever were all associated with aspergillus endocarditis [6] and thus are all clinical situations in which a broad differential is particularly important to consider.

In retrospect, it is possible that the culture-negative prosthetic valve endocarditis this patient initially presented with may have been due to Aspergillus, as was ultimately discovered during his final admission. Thus, it is possible that further workup with serologic testing or holding blood cultures for an extended period of time may have expedited his diagnosis. Several diagnostic modalities show promise in identifying the underlying etiology of culture-negative endocarditis patients. For example, Serum *Aspergillus Galactomannan* antigen is typically cleared by neutrophils; hence it is used to detect the presence of invasive aspergillosis in neutropenic patients; however, it has also been shown to identify the presence of disease in non-neutropenic patients as demonstrated in our patient [6]. Other studies suggest 18-fluorine-fluorodeoxyglucose positron emission tomography, Multi-detector Computed Tomography or a combination of the two modalities may be good diagnostic tools in patients with infective endocarditis, especially for prosthetic valves [7,8].

## CONCLUSION

Culture-negative prosthetic valve endocarditis presents unique diagnostic and therapeutic challenges. As with our case, there are rare cases of slow-growing organisms (Coxiella burnetii, *Bartonella, Legionella, pneumophilia, Brucella, Mycoplasma, Tropheryma whipplei, Escherichia. Coli, Streptococcus gallolyticus, Enterococci*, or fungal organisms) that can lead to months of negative cultures, non-diagnostic echocardiography, and non-definitive Duke criteria. In these difficult cases, a high index of suspicion is required, and less common diagnostic studies may provide a more timely diagnosis required for patient survival.

## Data Availability

The data and supportive information are available within the article.
